# Prevalence of back and neck pain in Germany. Results from the BURDEN 2020 Burden of Disease Study

**DOI:** 10.25646/7855

**Published:** 2021-03-10

**Authors:** Elena von der Lippe, Laura Krause, Michael Porst, Annelene Wengler, Janko Leddin, Anja Müller, Marie-Luise Zeisler, Aline Anton, Alexander Rommel, BURDEN 2020 study group

**Affiliations:** Robert Koch Institute, Berlin Department of Epidemiology and Health Monitoring

**Keywords:** BACK PAIN, NECK PAIN, BURDEN OF DISEASE, COMORBIDITIES, ADULTS

## Abstract

Back and neck pain are widespread and can significantly reduce quality of life. A cross-sectional telephone survey (N=5,009) was carried out between October 2019 and March 2020 to gain a valid estimate of the prevalence of back and neck pain among adults in Germany. In addition to the frequency and intensity of back and neck pain, the study collected information about quality of life and comorbidity. The findings showed that 61.3% of respondents reported back pain in the last twelve months. Lower back pain was reported about twice as often as upper back pain, with 15.5% of respondents stating that they experienced chronic back pain. 45.7% reported neck pain, and 15.6% of respondents have experienced lower and upper back pain in addition to neck pain in the past year. Women are affected by all types of pain more often than men. About half of the respondents categorise their back or neck pain as moderate; older respondents report significantly more pain episodes per month than younger respondents. The results described here provide a comprehensive picture of the population-related limitations associated with back and neck pain and are used within the framework of the BURDEN 2020 study to quantify key indicators of burden of disease calculation.

## 1. Introduction

Back pain can have various causes [1] but is most often triggered by poor posture and damage to or illnesses of the bones, joints, connective tissues, muscles and nerves. Back pain accompanied by clear medical evidence of a cause is referred to as specific back pain [2], whereas pain without a clear cause is referred to as non-specific back pain. Non-specific back pain is usually the result of a complex combination of psychological, social and biophysical factors [3–6] and is far more common than specific back pain [2, 7].

Although hardly any data exist on the prevalence of neck pain among the population in Germany [8], the importance of back pain has been well documented. Almost two thirds of the general population in Germany suffers from back pain every year, with around one fifth reporting chronic back pain (back pain that occurs almost daily for three months or more) [9–11]. Depending on its intensity and duration, back pain can be associated with limitations in daily activities, reductions in health-related quality of life and an increased risk of mental disorders such as depression and anxiety [12, 13]. Back pain is also one of the most common reasons for utilising the health system, taking sick leave, and opting for early retirement [1, 14, 15]. Data from the health insurer AOK from 2017 show that back pain (International Statistical Classification of Diseases and Related Health Problems, 10th Revision (ICD-10): M54) was the second most common reason for a sick leave among the employed health insured persons: back pain was associated with 6.1% of sick leave and 6.1% of cases of sickness-related incapacity to work [16].

The public health relevance of back pain becomes particularly evident when health-related quality of life is considered. Reduced quality of life due to disease-related limitations is measured as part of calculations of burden of disease based on years of healthy life lost [17]. In 2017, the Global Burden of Disease study found that 14.3% of years lived with disability (YLD) in Germany were caused by lower back pain. The study ranked it number one out of all conditions, with neck pain in third place at 5.0% [18]. However, the Global Burden of Disease study faces a challenge due to the limited availability of data at the regional level with which to estimate burden of disease [19–21]. This demonstrates the importance of integrating the collection of information on pain disorders into national health surveys in order to improve the data available with which to calculate national disease burden.

The ‘BURDEN 2020 – The burden of disease in Germany at the national and regional level’ study calculate years of life lost (YLD) for important illnesses and conditions, including back and neck pain. As such, the study also aimed to improve the data basis for calculations of national disease burden and to adapt it to specific informational needs.

This article is based on a survey carried out by the Robert Koch Institute (RKI) and aims to provide a detailed overview of back and neck pain among adults in Germany. It differentiates between lower and upper back pain, and, therefore, closes data gaps in Germany by providing information about pain localisation and the overlap between different types of pain. Information on frequency is supplemented by data on the frequency of pain episodes, the intensity of pain and the resulting limitations in everyday activities.

## 2. Methodology

### 2.1 Data collection

A nationwide cross-sectional telephone study of head, back and neck pain was conducted in Germany between October 2019 and March 2020. The survey covered German-speaking individuals aged 18 or above who were resident in Germany. Sampling and fieldwork are described in detail elsewhere [22]. The study collected detailed information about the characteristics of individual pain disorders and levels of general health and life satisfaction. A total of 5,009 respondents took part in the study (unweighted numbers): 2,634 women (52.6%) and 2,375 men (47.4%) [22]. The response rate, which was calculated using the criteria drawn up by the American Association for Public Opinion Research (AAPOR), was 24.0% [23].

### 2.2 Indicators

**Figure 1:**
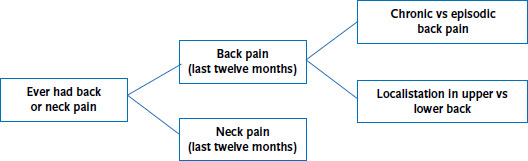
Schematic representation of the collection of data on back and/or neck pain Source: Study of head, back and neck pain in Germany (2019/2020)

In order to ensure a high degree of comparability, data on back and neck pain were collected using established instruments that are regularly deployed in surveys undertaken by the RKI [24]. Participants were asked whether they had ever experienced neck or back pain. The study focused on respondents who reported neck or back pain in the twelve months before the survey (Figure 1). The obtained data can also be used to draw conclusions about back pain localisation. The participants were asked whether their pain was located in the upper back (the thoracic spine), in the lower back (the lumbar spine, in the buttocks or in the hips) or in both areas. The study defined chronicity in line with the definition used by the German Society for General Medicine and Family Medicine and the National Care Guideline ‘Non-specific lower back pain’ as almost daily back pain that persists for three months or more [25]. Non-chronic back pain was defined as episodic back pain. In order to gain a more in-depth picture of back and neck pain, the participants were asked about the intensity and frequency of their pain. Moreover, people who experienced back or neck pain were asked to classify their pain as mild, moderate, severe or very severe. Pain frequency was defined as the number of days a person had experienced back pain during the past twelve months. People with back pain could also indicate whether their pain radiated into their legs.

### 2.3 Statistical analysis

In order to ensure that the results from the sample can be viewed as representative of the population in Germany, both the selection probability of an individual person (design weighting) and the population structure (adjustment weighting) were taken into account. Design weighting was aimed at controlling for the sample design and used a method to determine the selection probability of telephone samples comprising both landline and mobile phone numbers (dual frame design) [26]. Adjustment weighting was carried out iteratively by sex, age, education and region in order to ensure that these characteristics were distributed in a comparable manner to the structure of the resident population aged 18 or over in Germany. Data from the Federal Statistical Office (population as of 31 December 2018) and the microcensus from 2017 served as a basis for comparison [27, 28].

Statistical relationships between various characteristics were also tested for. The Pearson χ^2^ test for survey samples was used for nominal data [29]. With regard to purely metric values, differences in mean values between groups were determined using the t-test [30]. A statistically significant difference is assumed if the corresponding p-value is smaller than 0.05. All analyses were carried out using STATA (StataCorp LLC, Texas, US) version 15.1. All of the results presented in this article, including those with 95% confidence intervals (95% CI), are weighted and were obtained using the survey procedures for complex samples.

## 3. Results

**Figure 2:**
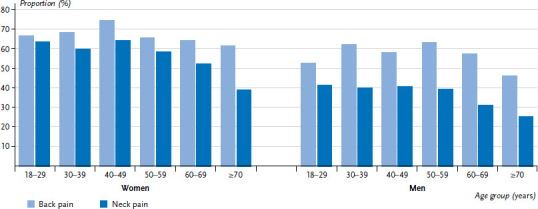
Proportion of adults with back or neck pain within the last twelve months by sex and age (n=2,634 women, n=2,375 men) Source: Study on head, back and neck pain in Germany (2019/2020)

61.3% of people in Germany reported back pain at least once in the past twelve months; 45.7% reported neck pain during the same period. Women (66%) report back pain significantly more often than men do (56.4%). A more pronounced difference was identified for neck pain with 54.9% of women and 36.2% of men stating that they experienced neck pain. Few significant differences were identified by age, with the exception of people aged 70 or over, who reported having experienced back and neck pain less often than the younger respondents (Figure 2, Annex Table 1).

The study also distinguished between pain in the lower back (lumbar spine and below) and the upper back (the thoracic spine). Lower back pain (52.9%) is about twice as common as upper back pain (27.4%) among the general population. Only minor differences were found by age (Annex Table 1). 38.5% of people with lower back pain report that their pain radiates into their legs.

**Figure 3:**
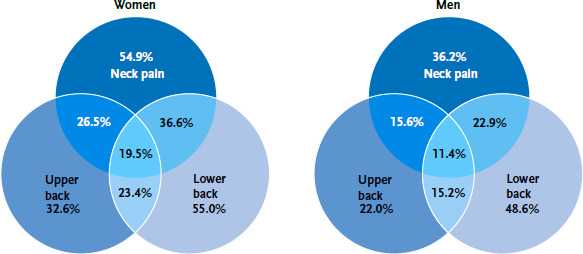
Proportion of adults with back or neck pain and comorbidities within the last twelve months by sex (n=2,634 women, n=2,375 men) Source: Study of head, back and neck pain in Germany (2019/2020)

Some respondents reported that they experienced pain in more than one part of their body. 19.4% reported both lower and upper back pain during the twelve months before the study. In addition, an overlap between lower back pain and neck pain was identified among 21.2% of respondents, and between upper back and neck pain among 29.9%. 15.6% of respondents stated that they had experienced pain in all three areas during the last twelve months. Women reported pain in all three areas of the back and the neck, as well as an overlap, significantly more often than men (Figure 3).

**Figure 4:**
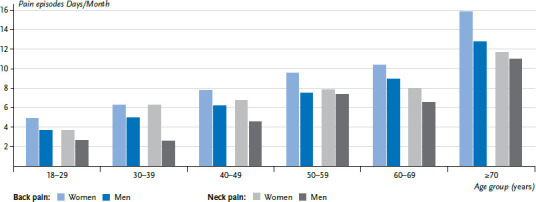
Average frequency of back and neck pain episodes in affected individuals by age and sex (days/month) (back pain: n=1,605 women, n=1,280 men; neck pain: n=1,289 women, n=810 men) Source: Study on head, back and neck pain in Germany (2019/2020)

The frequency of pain episodes increases significantly with age. Whereas participants aged between 18 and 29 with back pain reported pain on an average of 4.4 days per month, the same participants aged 70 or above reported back pain on an average of 14.8 days. In addition, the frequency of neck pain increases with age from an average of 3.3 days to 11.5 days per month. On average, women affected by back pain experience back pain on 9.3 days and men on 7.2 days per month. Similarly, women with neck pain experience pain on 7.2 days a month, compared with 5.4 days for men. The differences identified between men and women are statistically significant. These differences remain largely constant with age, the age-related increase in the number of days with pain episodes is equal for both sexes (Figure 4).

In line with the age-associated increase in the frequency of pain episodes, chronic back pain increases significantly with age. Although chronic back pain is still relatively rare (4.5%) among people aged between 18 and 29, almost a quarter of people aged 70 or over (23.4%) suffer from chronic back pain (Annex Table 1). The prevalence of chronic back pain in the population is 15.5% and is significantly higher among women (18.5%) than men (12.4%). Among people with back pain, 33.9% of women and 25.9% of men have chronic back pain. Consequently, around two thirds of women with back pain and three quarters of men with back pain report episodic pain.

**Figure 5:**
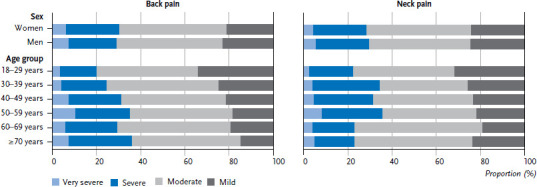
Intensity of back and neck pain by age and sex (Back pain: n=1,605 women, n=1,280 men; neck pain: n=1,289 women, n=810 men) Source: Study on head, back and neck pain in Germany (2019/2020)

Comparatively few differences between men and women were identified for pain intensity. Overall, 23.2% of people with back pain report severe pain and 6.4% report very severe pain. 24.0% of people with neck pain report severe pain and 4.6% very severe pain. However, although hardly any differences were found between men and women with regard to pain intensity (Figure 5), a clear association was identified between severity of back pain and age. 20.0% of participants aged between 18 and 29 who experienced back pain report severe or very severe pain, this applies to 36.0% of people aged 70 or above.

## 4. Discussion

61.3% of respondents reported back pain and 45.7% reported neck pain in the twelve months before the survey. Lower back pain is about twice as common as upper back pain. 15.6% of respondents reported lower and upper back pain in addition to neck pain in the past twelve months. 15.5% of respondents reported chronic back pain. Women report back and neck pain and also more pain episodes per month than men. Chronic back pain is also more common among women. However, there are only slight differences between men and women with regard to perceptions of back pain intensity. Overall, more than two-thirds of respondents reported at least moderate pain, with older respondents reporting severe or very severe pain more often and more pain episodes per month than younger respondents.

When comparing the results presented here to the literature, it is important to take into account the fact that studies of pain often examine different age groups and time intervals, and differ in the way that they operationalise pain in terms of intensity, frequency, localisation and type of overlap [8–11, 31–39]. In addition, sociocultural aspects play a role in the perception and assessment of pain, and, therefore, can also have an impact on pain prevalence [40]. In this respect, studies from Germany that consider neck pain and (chronic) back pain in the last twelve months are particularly relevant for comparison.

An analysis based on data from the 1998 Federal Health Survey carried out by the RKI showed that at the time of the survey, 44.8% of those questioned had suffered from neck pain and 59.4% from back pain in the previous twelve months [8]. In an analysis based on data from the German Telephone Health Survey 2003 (GSTel03), which was conducted by the RKI, 61.8% of participants reported back pain in the twelve months before the survey [9]. More comparative studies are available for Germany when it comes to the twelve-month prevalence of chronic back pain. The German Health Update (GEDA) studies, which were carried out in several waves by the Robert Koch Institute, [41, 42] found that around one fifth of respondents reported chronic back pain (pain persisting for at least three months and occurring almost daily) [9–11, 37]. Similarly, the 2017 NAKO health study found that 22.5% of people reported chronic back pain [37]. Overall, the figures presented here are similar to previous studies, albeit somewhat lower in the case of chronic back pain. Although data on multilocalisation are gathered differently by various studies, which makes direct comparisons impossible, [38, 39] the literature confirms the finding that many affected individuals experience pain in different locations at the same time.

Other studies have also found that women report neck pain and (chronic) back pain more frequently than men [6, 9–11, 37]. There are probably various reasons for this [9, 43]: in addition to anatomical differences such as muscle strength, women often perceive their body differently from the way men do and tend to react more sensitively to pain [9, 43]. It is also possible that differences in cerebral pain processing and hormonal differences in the perception of pain also play a role [44].

This study provides up-to-date and reliable information on back and neck pain in Germany. The sample design, the large number of participants (over 5,000) and the weighting used for the analysis ensure that the study is representative of the German-speaking general population. As this study distinguishes between back and neck pain and between lower and upper back pain, it also provides information that is missing in many other population-based studies. Since recurring back and neck pain can be burdensome for some of the people it affects, it is fair to assume that they can judge the severity of the pain they experience. The smooth manner in which the fieldwork was undertaken, the small proportion of missing values and the plausibility of the findings suggest that the participants also understood the questions.

In terms of limitations, it is important to mention that this study was impacted by a general decreasing willingness to participate in studies. Besides, the response rate is comparable to that of other studies conducted by the RKI, and elsewhere [42]. Although the data set was adjusted to correct for deviations from the population in terms of socio-demographic characteristics, it remains unclear whether, for example, chronically ill people were adequately represented and, if they were not, what impact this might have on the prevalence of back and neck pain.

### 4.1 Outlook

Some people experience back and neck pain as burdensome and it can result in a severe reduction in quality of life. The importance of pain disorders for the health of the population is particularly evident from studies of disease burden. As part of the BURDEN 2020 study, a primary survey was chosen to quantify the prevalence and severity distribution of pain disorders as part of a national Burden of Disease study. This approach has proven successful for back and neck pain, as before for migraine and tension-type headache [22]. Moreover, it enabled the approach used by the Global Burden of Disease Study to be implemented appropriately. The results presented here illustrate the need for early prevention and care. The frequency and intensity of back and neck pain increases sharply with age, which itself is associated with a growing burden of disease. In order to prevent this development, it is important to offer preventive approaches such as exercise and work-related measures as well as promising health care approaches such as multimodal therapy and to do so broadly while ensuring they are implemented effectively at a young age.

## Key statements

- 61.3% of respondents reported back pain in the last twelve months; 45.7% reported neck pain.
- Around one sixth of respondents (15.5%) suffers from chronic back pain.
- Women report back pain and neck pain (66.0%, 54.9%) more often than men do (56.4%, 36.2%).
- On average, back pain is experienced on 8.3 days per month and neck pain on 6.5 days.
- Lower back pain (52.9%) is about twice as common as upper back pain (27.4%).

## Appendix

**Annex Table 1:** Twelve-month prevalence of back and neck pain by age and sex (n=2,634 women, n=2,375 men) Source: Study on head, back and neck pain in Germany (2019/2020)

Age group	Back pain				Upper back pain				Lower back pain			
	Women		Men		Women		Men		Women		Men	
	%	(95% CI)	%	(95% CI)	%	(95% CI)	%	(95% CI)	%	(95% CI)	%	(95% CI)
18–29 years	66.2	(56.6–74.6)	52.3	(44.5–60.0)	33.7	(25.4–43.1)	26.5	(20.2–34.0)	48.9	(39.3–58.5)	42.0	(34.6–49.8)
30–39 years	67.8	(59.5–75.0)	61.9	(52.8–70.2)	42.2	(34.6–50.3)	30.8	(22.9–40.1)	57.3	(49.2–65.1)	51.1	(42.1–60.1)
40–49 years	73.9	(67.3–79.5)	57.7	(49.1–65.8)	34.4	(28.0–41.5)	24.1	(17.0–32.9)	60.9	(53.7–67.6)	50.3	(41.7–58.9)
50–59 years	65.3	(59.4–70.7)	62.8	(56.3–68.9)	34.1	(28.5–40.2)	22.3	(17.4–28.1)	54.6	(48.6–60.4)	57.0	(50.4–63.3)
60–69 years	63.9	(57.9–69.6)	57.1	(50.6–63.4)	34.1	(28.3–40.3)	14.0	(10.4–18.5)	53.6	(47.5–60.0)	49.4	(42.9–55.9)
≥70 years	61.2	(55.0–67.0)	45.8	(39.2–52.5)	21.2	(16.5–26.9)	13.3	(9.2–19.0)	55.0	(49.0–61.0)	40.8	(34.5–47.5)
**Total**	**66.0**	**(63.1–68.7)**	**56.4**	**(53.3–59.4)**	**32.6**	**(29.8–35.4)**	**22.0**	**(19.4–24.8)**	**55.0**	**(52.0–57.9)**	**48.6**	**(45.5–51.7)**

Age group	Chronic back pain^1^				Neck pain			
	Women		Men		Women		Men	
	%	(95% CI)	%	(95% CI)	%	(95% CI)	%	(95% CI)
18–29 years	6.3	(3.1–12.7)	2.8	(1.1–7.0)	63.2	(53.6–71.8)	41.0	(33.6–48.8)
30–39 years	11.8	(7.5–18.0)	10.8	(5.5–20.0)	59.5	(51.4–67.1)	39.7	(31.4–48.7)
40–49 years	19.1	(13.2–26.9)	11.9	(7.3–18.8)	63.6	(56.5–70.2)	40.3	(32.0–49.1)
50–59 years	21.9	(16.6–28.5)	15.9	(10.9–22.6)	57.8	(52.0–63.5)	38.9	(32.8–45.4)
60–69 years	20.7	(15.8–26.6)	16.7	(11.5–23.7)	52.0	(45.9–57.9)	30.9	(25.3–37.1)
≥70 years	28.0	(22.2–34.6)	17.4	(12.6–23.5)	38.9	(33.1–45.0)	25.3	(19.6–32.0)
**Total**	**18.5**	**(16.2–21.1)**	**12.4**	**(10.3–14.9)**	**54.9**	**(52.0–57.8)**	**36.2**	**(33.3–39.2)**

^1^ Back pain that occurs almost every day and persists for three months or more

CI = confidence interval
